# Developing a comprehensive measure of mobility: mobility over varied environments scale (MOVES)

**DOI:** 10.1186/s12889-017-4450-1

**Published:** 2017-05-25

**Authors:** Jana A. Hirsch, Meghan Winters, Joanie Sims-Gould, Philippa J. Clarke, Nathalie Ste-Marie, Maureen Ashe, Heather A. McKay

**Affiliations:** 1 Department of Epidemiology and Biostatistics, Arnold School of Public Health, University of South Carolina, 915 Greene Street, Columbia, SC USA; 2 Centre for Hip Health and Mobility, Robert H.N. Ho Research Centre, University of British Columbia, 5th Floor, 2635 Laurel St, Vancouver, BC Canada; 3 Faculty of Health Sciences, Simon Fraser University, 8888 University Drive, Burnaby, BC Canada; 4 Department of Family Practice, University of British Columbia, 3rd Floor David Strangway Building, 5950 University Boulevard, Vancouver, BC Canada; 5 Department of Epidemiology, University of Michigan, 1415 Washington Heights, 4667 SPH I, Ann Arbor, MI USA; 6 Institute for Social Research, University of Michigan, P.O. Box 1248, 426 Thompson St, Ann Arbor, MI USA; 7 Division of Orthopaedic Surgery, McGill University Health Centre, 1001 Boulevard Décarie, Montréal, QC Canada; 8 Department of Orthopaedics, University of British Columbia, 3114 - 910 West 10th Avenue, Vancouver, BC Canada

**Keywords:** Mobility limitation, Measurement, Methods, Functionally-Impaired elderly, Aged, Elderly, Surveys and questionnaires, Social interaction, Transportation

## Abstract

**Background:**

While recent work emphasizes the multi-dimensionality of mobility, no current measure incorporates multiple domains of mobility. Using existing conceptual frameworks we identified four domains of mobility (physical, cognitive, social, transportation) to create a “Mobility Over Varied Environments Scale” (MOVES). We then assessed expected patterns of MOVES in the Canadian population.

**Methods:**

An expert panel identified survey items within each MOVES domain from the Canadian Community Health Survey- Healthy Aging Cycle (2008–2009) for 28,555 (weighted population *n* = 12,805,067) adults (≥45 years). We refined MOVES using principal components analysis and Cronbach’s alpha and weighted items so each domain was 10 points. Expected mobility trends, as assessed by average MOVES, were examined by sociodemographic and health factors, and by province, using Analysis of Variance (ANOVA).

**Results:**

MOVES ranged from 0 to 40, where 0 represents individuals who are immobile and 40 those who are fully mobile. Mean MOVES was 29.58 (95% confidence interval (CI) 29.49, 29.67) (10th percentile: 24.17 (95% CI 23.96, 24.38), 90th percentile: 34.70 (CI 34.55, 34.85)). MOVES scores were lower for older, female, and non-white Canadians with worse health and lower socioeconomic status. MOVES was also lower for those who live in less urban areas.

**Conclusions:**

MOVES is a holistic measure of mobility for characterizing older adult mobility across populations. Future work should examine individual or neighborhood predictors of MOVES and its relationship to broader health outcomes. MOVES holds utility for research, surveillance, evaluation, and interventions around the broad factors influencing mobility in older adults.

**Electronic supplementary material:**

The online version of this article (doi:10.1186/s12889-017-4450-1) contains supplementary material, which is available to authorized users.

## Background

While the pace and pattern of population shifts differ across the world, the older population is increasing globally [1]. In North America the proportion of the population 65 years and older is expected to rise from 12.8% in 2008 to 20.8% in 2040 [1]. This unprecedented shift demands that systems and communities meet needs of this aging demographic. Mobility restrictions influence older adult independence [2], constrict community engagement [3, 4], and increase negative health outcomes and premature mortality [5, 6]. Thus it is imperative that we devote collective attention to strategies and tools that support maintaining mobility later in life.

Mobility is multi-dimensional and includes the importance of social and community engagement, use of transportation, and cognition [7, 8]. The Canadian Institute for Health Research (CIHR) acknowledged this broader definition of mobility; in the Mobility in Aging Strategic Initiative (CIHR Institute of Aging) mobility was defined as encompassing participation in society, as well as the ability to drive and access public transportation [9]. In the transportation realm, mobility is often measured as trip rate (any mode). In addition, transportation studies recognized that mobility should include one of the following dimensions: 1) access to places of desire (such as visiting family or friends), 2) psychological benefits of travel (either social contact or independence), or 3) benefits of physical movement itself and potential travel [10–12]. Urban planning recognized community environments as important in shaping mobility [13–20]. Understandably, advocacy groups focused on the role neighborhoods play in maintaining independence and mobility for older adults [21, 22].

Methods used to assess mobility vary across research studies and fields [7]. However, existing metrics often focus on an individual’s capacity for, or enacted physical function. Cognitive ability to engage, social connections with an older person’s community, or transportation choices are most often excluded from these metrics. Current measures of mobility include assessments of transfer skills, gait, or wheelchair mobility [23–25]. Activities of daily living (ADL) and instrumental activities of daily living (IADL) are also used to assess mobility clinically [26, 27]. These methods were criticized as failing to capture what people actually do in their daily lives [28] or how an individual is involved in social situations [29]. Life-space measures attempt to capture broader mobility, by including mobility inside the home, outside the home, within the neighborhood, and beyond [28]. Yet the life-space measure does not capture transportation patterns or community engagement of older adults directly. Given the expanding definition of mobility, and the importance of mobility for older adults, there is a need for measures of mobility that encompass these domains. Therefore, we respond to both the opportunity and need for a holistic measure of older adult mobility that includes physical, cognitive, social, and transportation domains.

Thus the objectives of our study were twofold: 1) to create a Mobility Over Varied Environments Scale (MOVES) using a large, population based study of Canadian older adults, and 2) to apply MOVES to examine the distribution of mobility across sociodemographic and health characteristics of the Canadian population. This second objective allows us to examine the performance of MOVES. For this, we hypothesize that MOVES will follow known patterns of mobility, including lower mobility for Canadians in worse health, at older ages, or with lower socioeconomic status.

### Conceptual frameworks

MOVES draws on the comprehensive mobility framework outlined by Webber et al. [7] and the World Health Organization’s *International Classification of Functioning, Disability, and Health (ICF)* [8]. Webber et al. defined mobility broadly as “the ability to move oneself (e.g., by walking, by using assistive devices, or by using transportation) within community environments that expand from one’s home, to the neighborhood, and to regions beyond.” This framework acknowledges that mobility takes many forms, including walking, using a wheelchair, driving, and using alternate forms of transportation. The Webber framework identifies five key domains that determine older adult mobility: physical, cognitive, psychosocial, environmental, and financial [7]. These domains are interrelated. For example, an individual’s physical impairments (physical) with or without accompanying psychological factors (e.g. depression) can contribute to the development of fear of falling (cognitive), leading to activity restriction and reduced social engagement (psychosocial). Similarly, the ICF has a broad description of mobility that captures both indoor and outdoor movement as well as the use of assistive devices and transportation. Further, the description includes participation in activities and environmental factors that play a role in mobility.

## Methods

### MOVES creation

**Fig. 1:**
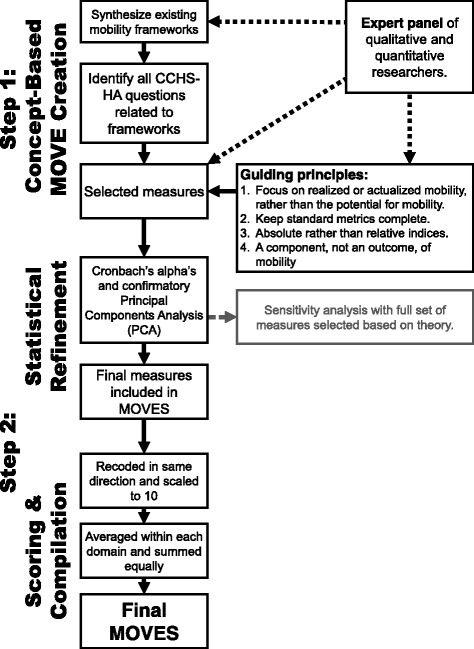
Iterative process to create the Mobility Over Varied Environment Scale (MOVES). Dotted lines indicate the involvement of an expert panel of qualitative and quantitative researchers who played three key roles: 1) helping to synthesize the mobility frameworks 2) selecting specific items based on questions identified in CCHS-HA and 3) establishing guiding principles for the creation of MOVES that were used to select specific items in CCHS-HA. Note that the creation of MOVES was primarily based in conceptual frameworks and then underwent statistical refinement to both confirm frameworks and tailor the MOVES measure. A sensitivity analysis was run including all items based on frameworks (had barriers and limitations within each domain as well as an additional financial domain)

We created MOVES based on the two conceptual frameworks outlined above. Its design was executed in an iterative process involving qualitative and quantitative researchers across multiple fields (Figure 1). The process had two broad steps: 1) concept-based creation of MOVES; and 2) statistical refinement, scoring and final compilation.

#### Concept-based MOVES creation

An expert panel of researchers and staff (*n* = 10) from gerontology, epidemiology, family medicine, transportation, and health behavior played a critical role in item selection. First, they helped synthesize existing mobility frameworks. Second, after two researchers separately identified items from the Canadian Community Health Survey- Healthy Aging (CCHS-HA) that related to the mobility frameworks, the expert panel determined which items to include.

#### Statistical refinement

On the selected items, we ran Cronbach’s alpha and a confirmatory Principal Component Analysis (PCA) to determine whether: 1) items were contributing to their respective domains and the overall score, 2) the items grouped together as anticipated, and 3) what proportion of variance was explained by these items. Items were the combined into a final MOVES.

### Understanding canadian mobility using MOVES

We applied MOVES to the CCHS-HA to better understand the distribution of mobility in the Canadian population. The CCHS-HA is a cross-sectional survey (*n* = 30,865) of the Canadian population living in the 10 provinces across Canada (Canadian territories were excluded). Details can be found elsewhere [30]. Briefly the Healthy Aging component was completed December 2008 through November 2009 and surveyed people (≥ 45 years) using computer assisted personal interviewing (94% of interviews conducted in person) achieving an overall response rate of 74.4%. For the creation of MOVES, we included CCHS-HA participants who had all component items that comprised MOVES (final *n* = 28,555).

Weighted frequencies were used to describe the sociodemographic characteristics of the CCHS-HA sample. MOVES mean score was examined across each sociodemographic characteristic. We obtained *p*-values for comparisons across categories from t-tests and analysis of variance (ANOVA). MOVES mean score was also compared across age and gender within each province. We weighted all results using the Statistics Canada proportional sampling scheme and applied Balanced Repeated Replication (BRR) with 500 bootstrap weight variables to obtain the correct standard errors for ANOVA. All analyses were conducted using SAS, version 9.4 (SAS Institute Incorporated: Cary, NC).

### Results- moves creation

#### Item selection

To select items*,* the expert panel established four guiding principles: 1) MOVES should focus on actualized or realized mobility of an individual, rather than potential for mobility (e.g. how often one engages in community activities versus whether community activities exist), 2) if there were existing metrics within a domain, these metrics should remain intact, rather than being split into their component parts, 3) where possible, MOVES should be an absolute rather than a relative metric, to be applicable beyond the Canadian population, and 4) items should represent components, rather than outcomes, of mobility (e.g. loneliness was excluded as it may result from low social engagement).

#### MOVES domains

In practice, the measurement of Webber’s psychosocial domain and cognitive domain overlap. Therefore, to develop MOVES we modified the psychosocial domain to be primarily social, based on the complementary domain from the ICF, “activities and participation.” This domain includes interpersonal interactions and relationships, as well as community social and civic life. Similarly, many of the environmental determinants in both Webber and ICF models are related to service systems and policies that influence transportation mode. Therefore, this domain was conceptualized more narrowly in our work as “transportation.”

#### Physical

Our expert panel identified eight items (five of which were barriers or limitations) to include in the physical domain (Table 1). We used activities of daily living (ADL), ambulation, and physical activity items to capture physical function and activity. ADL items excluding meal preparation come from the Older Americans Resources and Services (OARS) Multidimensional Functional Assessment Questionnaire© (OMFAQ) [31]. Ambulation items were from the adapted version of the Health Utilities Index (HUI) mark 3 [32], a validated instrument which provides a description of an individual’s overall functional health. Because sedentary behavior and physical activity independently predict successful aging [33], physical activity was measured using the Physical Activity Scale for the Elderly (PASE), a validated and copyrighted instrument (1991) developed by the New England Research Institutes (NERI) to provide an overall assessment of self-reported occupational, household and leisure activities over the past seven days in older persons [34]. Barriers and limitations included reporting a health condition limiting participation in activities, public transportation use, or health improvements.

**Table 1:** Full set of component items for each domain of the MOVES included in both the final MOVES score and sensitivity analysis

Item	Points toward MOVES^a^	Weighted Percent of responses (95% CI)
Physical Domain		
Instrumental & Basic Activities of Daily Living Classification		
No Functional Impairment	10	89.1 (88.5, 89.6)
Mild Impairment	7.5	7.9 (7.4, 8.4)
Moderate Impairment	5	2.1 (1.8, 2.3)
Severe Impairment	2.5	0.5 (0.4, 0.6)
Total Impairment	0	0.5 (0.4, 0.6)
Ambulation (Mobility)		
Able to walk around the neighbourhood without difficulty, and without walking equipment	10	93.4 (93.0, 93.8)
Able to walk around the neighbourhood with difficulty; but does not require walking equipment or the help of another person	8	1.2 (1.0, 1.4)
Able to walk around the neighbourhood with walking equipment, but without the help of another person	6	3.7 (3.4, 4.0)
Able to walk only short distances with walking equipment, and requires a wheelchair to get around the neighbourhood	4	0.3 (0.2, 0.3)
Unable to walk alone, even with walking equipment. Able to walk short distances with the help of another person, and requires a wheelchair to get around the neighbourhood	2	1.1 (0.9, 1.2)
Cannot walk at all	0	0.3 (0.3, 0.4)
Physical Activity Scale for the Elderly (PASE) Score		
Quartile 1	10	25.1 (24.0, 26.1)
Quartile 2	6.67	25.1 (24.1, 26.1)
Quartile 3	3.33	25.1 (24.1, 26.0)
Quartile 4	0	24.8 (23.9, 25.7)
Reported that health condition limited participation in (more) activities^b^	−1	5.8 (5.4, 6.2)
Reported that health condition limited use of public transportation^b^	−1	2.0 (1.8, 2.2)
Reported that health condition limited use of accessible transportation^b^	−1	0.5 (0.4, 0.5)
Reported that physical condition is a barrier to improve health^b^	−1	2.8 (2.5, 3.1)
Reported that disability or health problem is a barrier to improve^b^	−1	3.6 (3.3, 4.0)
Cognitive Domain		
Cognition		
Able to remember most things, think clearly and solve day to day problems	10	73.9 (73.0, 74.9)
Able to remember most things, but have a little difficulty when trying to think and solve day to day problems	8	2.2 (1.9, 2.5)
Somewhat forgetful, but able to think clearly and solve day to day problems	6	17.2 (16.3, 18.0)
Somewhat forgetful, and have a little difficulty when trying to think or solve day to day problems	4	5.0 (4.6, 5.5)
Very forgetful, and have great difficulty when trying to think or solve day to day problems	2	1.4 (1.2, 1.6)
Unable to remember anything at all, and unable to think or solve day to day problems	0	0.2 (0.1, 0.3)
Fear of falling		
Not applicable (<65 years old)	10	68.1 (67.2, 68.9)
Not worried or concerned about future falls	10	21.2 (20.5, 21.9)
Worried or concerned about future falls, have not stopped activities	5	6.0 (5.6, 6.3)
Worried or concerned about future falls, have stopped some activities	0	4.8 (4.5, 5.0)
Transport Domain		
Number of modes (comprised of the modes below)^c^		
No Modes	0	0.3 (0.2, 0.4)
1 Mode	2.5	30.2 (29.2, 31.2)
2 Modes	5	36.7 (35.6, 37.8)
3 Modes	7.5	25.4 (24.4, 26.3)
4 Modes	10	7.5 (6.8, 8.1)
Modes of transport used in past month^c^		
Drive (at least once in past week)	2.5	83.0 (82.3, 83.7)
Passenger (passenger/taxi)	2.5	64.5 (63.4, 65.6)
Transit (public transit/accessible transit)	2.5	21.9 (21.0, 22.9)
Active travel (cycling/walking)	2.5	40.2 (39.1, 41.3)
Reported that transportation problems limited participation in (more) activities^b^	−1	1.3 (1.1, 1.5)
Reported that transportation problems is a barrier to improve health^b^	−1	0.2 (0.1, 0.3)
Reported that transportation problems are the reason they did not see the dentist^b^	−1	0.0 (0.0, 0.1)
Social Domain		
Sense of belonging to local community		
Very strong	10	22.1 (21.2, 23.0)
Somewhat strong	6.67	44.0 (42.8, 45.1)
Somewhat weak	3.33	23.4 (22.5, 24.4)
Very weak	0	10.1 (9.5, 10.8)
Frequency of participation in a community-related activity		
Did not participate in a community-related activity	0	1.9 (1.7, 2.1)
Participated at least once a year	2.5	4.5 (4.0, 4.9)
Participated at least once a month	5	20.8 (19.8, 21.7)
Participated at least once a week	7.5	60.9 (59.8, 62.0)
Participated at least once a day	10	11.9 (11.2, 12.7)
Tangible social support (higher values indicate higher social support)		
Index score from 0 to 16	0	0.8 (0.6, 1.0)
1	0.625	0.4 (0.3, 0.6)
2	1.25	0.6 (0.4, 0.7)
3	1.875	0.7 (0.6, 0.9)
4	2.5	1.2 (0.9, 1.4)
5	3.125	1.0 (0.8, 1.1)
6	3.75	1.4 (1.1, 1.6)
7	4.375	1.7 (1.4, 1.9)
8	5	3.1 (2.7, 3.4)
9	5.625	2.6 (2.3, 2.9)
10	6.25	3.8 (3.4, 4.2)
11	6.875	4.3 (3.9, 4.7)
12	7.5	10.9 (10.1, 11.7)
13	8.125	6.4 (5.9, 7.0)
14	8.75	8.3 (7.7, 9.0)
15	9.375	9.9 (9.2, 10.6)
16	10	43.0 (41.8, 44.1)
Financial Domain^d^		
Reported that cost limited participation in (more) activities^b^	−1	3.2 (2.8, 3.6)
Reported that cost limited use of public transportation^b^	−1	0.6 (0.4, 0.8)
Reported that cost limited use of accessible transportation^b^	−1	0.3 (0.2, 0.5)
Reported that cost is a barrier to improve health^b^	−1	1.5 (1.3, 1.7)

^a^All component items coded so that higher points indicate more positive mobility and then scaled to be between 0 and 10 points. Barriers each coded as penalties of 1 point

^b^Statistical refinement using Cronbach’s alpha identified that barrier and limitation items (including all of those in the financial domain) were not adding to the overall MOVES or the domains. These items only used in sensitivity analyses

^c^Number of modes was used for the MOVES, but the breakdown of each transport mode is also presented for descriptive purposes

^d^The Financial Domain was not included in the final MOVES due to results of the statistical refinement

#### Cognitive

In the psychological and cognitive domain, we used two items, one for cognition and one that measured fear of falling. Cognition was captured with the HUI cognitive health status [32]. This measures whether a respondent can remember most things, think clearly, and solve day-to-day problems. We used fear of falling to tap into self-efficacy around mobility. A survey item related to fear of falling was administered to all those 65 years or older (response categories: not worried or concerned, worried or concerned but haven’t stopped activities, and worried or concerned and have stopped activities).

#### Transportation

Transportation was measured using four items, one represented travel mode of the respondent and three reported transportation-related barriers and limitations. For travel mode, participants answered the question, “in the past month, which of the following (other) forms of transportation have you used?” Respondents were given the options: passenger in a motor vehicle; taxi; public transportation such as bus, rapid transit, subway or train, accessible transit, cycling, walking, wheelchair or motorized cart, or none. Barriers and limitation included reporting transportation problems that limited their participation or ability to improve their health.

#### Social

Social aspects of mobility were measured using three items: a sense of belonging to the local community; frequency of participation in community activities; and tangible social support. Sense of belonging was measured by asking respondents “How would you describe your sense of belonging to your local community? Frequency of community-related activity participation was assessed by participation in any type of community-related activity during the previous 12 months and then categorized as participation once a year, once a month, once a week, or once a day. Tangible social support was taken from the Medical Outcomes Study (MOS) Social Support Survey [35]. This scale ranges from 0 to 16 and was not asked during proxy interviews; therefore proxy respondents do not have a MOVES score.

#### Financial

The expert panel identified that an individual’s financial standing influences and interacts with the other domains. However, since income or wealth are not actualized mobility, we only included financial markers of whether an individual felt cost prohibited them from being mobile or engaging with their community (barriers and limitations). Ultimately, this domain was not included in MOVES due to findings during the statistical refinement process described below.

### Statistical refinement, scoring and compilation

We ran Cronbach’s alpha to determine internal consistency for all items (0.61) and within each domain (range from 0.11–0.64). By examining Cronbach’s alpha if each item were deleted, we identified that MOVES performed equally well without barrier and limitation items (including all those in the financial domain). After removing these items, the final MOVES standardized Cronbach’s alpha was 0.58. The cognitive domain had the lowest internal consistency, likely because cognitive function and fear of falling tap into different, yet related, elements of mobility-related cognition or psychology.

We ran PCA both on all items identified by the expert panel and just on items remaining after Cronbach’s alpha analysis. We ran these PCA with no restrictions placed on number of factors as well as with factor constraints equal to the number of domains. In general, items grouped within the anticipated domains. However, fear of falling loaded onto both the cognitive and physical domain and transportation mode loaded onto a number of factors. This cross-over between factors was expected, as theoretical frameworks include interconnectedness between domains. PCA also confirmed we should restrict to only four domains; in the solution including all items, the first five factors accounted for only 39.1% of variance (all five had eigenvalues greater than 1). In the solution using only the subset of items indicated by Cronbach’s alpha, the first four factors accounted for 62.3% of the variance (only the first three had eigenvalues greater than 1). Thus, statistical refinement using PCA confirmed that removing barriers and limitations (including the entire financial domain) from MOVES created an equally sound score.

We provide final items and scoring for items in MOVES in Table 1. All items (except PASE) were categorical and were left in their original metrics based on the guiding principle for absolute versus relative items. Scores were recoded so higher values indicate greater mobility and then were scaled to 10 points. As recommended by Statistics Canada [30], PASE data were used as quartiles. Since respondents aged under 65 were not asked about their fear of falling, we allocated them 10 points. We chose to allocate points based on the number of transportation modes each respondent reported. We did not prioritize active mode, aligned with the conceptual frameworks that considered all forms of transportation as important to mobility. We grouped transportation modes as: driving oneself (having a driver’s license and driving at least once in the previous month), being driven (being a passenger or taking a taxi), taking public or accessible transit (where accessible transit included service designed for persons with disabilities or mobility issues), and active transit (walking or cycling for transportation). Items within each domain were averaged, so each domain received an equal weight of 10 points. The final MOVES was created by summing across four domains for a possible score of 0 to 40.

## Results

### Canadian mobility

#### CCHS sample

In the weighted sample, 49% were female, most were between ages 45 and 64 (Table 2). A majority were married, white, Canadian, still working, have post-secondary education, own their homes, live in single detached houses, and live with their family. Most do not receive home care, were satisfied or extremely satisfied with life, have at least one chronic condition, drink regularly, do not have arthritis, have experienced no falls, and did not feel depressed or lose interest in things (Table 3).

**Table 2:** Distribution of MOVES across sociodemographic and economic characteristics

Sociodemographic or Economic Characteristics	Weighted Percentage (95% CI)	Mean MOVES (95% CI)	*p*-value for MOVES^a^
Sex			<.0001
Male	44.9 (43.7, 46.0)	29.9 (29.8, 30.0)	
Female	49.0 (47.9, 50.2)	29.3 (29.1, 29.4)	
Age			<.0001
45–54	36.7 (35.4, 37.9)	30.8 (30.6, 30.9)	
55–64	28.0 (27.1, 28.9)	30.4 (30.2, 30.5)	
65–74	16.6 (16.0, 17.2)	28.5 (28.3, 28.6)	
75–84	9.7 (9.3, 10.1)	26.5 (26.3, 26.7)	
85+	3.0 (2.8, 3.2)	24.0 (23.7, 24.3)	
Worked at job or business			<.0001
Yes	57.3 (56.2, 58.3)	31.0 (30.8, 31.1)	
No	38.8 (37.8, 39.8)	27.6 (27.4, 27.7)	
Retirement status			<.0001
Completely retired	33.0 (32.0, 33.9)	27.9 (27.8, 28.0)	
Partially retired or not retired	61.7 (60.7, 62.7)	30.7 (30.6, 30.8)	
Highest level of education			<.0001
Less than or secondary school graduation	24.6 (23.7, 25.5)	27.9 (27.8, 28.1)	
Some post-secondary	4.5 (4.1, 5.0)	29.7 (29.3, 30.1)	
Post-secondary graduation	64.9 (63.9, 65.9)	30.2 (30.1, 30.3)	
Total household income from all sources			<.0001
Less than $19,999	9.1 (8.6, 9.6)	26.3 (26.0, 26.5)	
$20,000 TO $49,999	28.3 (27.3, 29.3)	28.5 (28.4, 28.6)	
$50,000 TO $99,999	33.8 (32.6, 35.0)	30.4 (30.2, 30.5)	
$100,000 and over	24.1 (22.9, 25.2)	31.5 (31.3, 31.7)	
Region or Province of residence			<.0001
Atlantic	7.2 (6.9, 7.5)	29.1 (28.9, 29.2)	
Quebec	23.2 (22.3, 24.2)	28.9 (28.7, 29.0)	
Ontario	36.4 (35.2, 37.6)	29.6 (29.4, 29.7)	
Prairies	14.6 (14.0, 15.2)	30.2 (30.1, 30.4)	
British Columbia	12.5 (11.8, 13.2)	30.5 (30.2, 30.7)	
Population size group			0.0033
Rural area	15.1 (14.2, 16.0)	29.2 (29.0, 29.4)	
Urban area < 100,000	13.4 (12.7, 14.2)	29.4 (29.2, 29.6)	
≥ 100,000 to <500,000	21.2 (20.3, 22.0)	29.5 (29.4, 29.7)	
≥ 500,000	44.2 (43.1, 45.3)	29.8 (29.6, 29.9)	
Flag for tenure of dwelling			<.0001
Not owned by the respondent	16.9 (16.1, 17.7)	27.8 (27.6, 28.0)	
Owned by the respondent	77.5 (76.6, 78.4)	30.0 (29.9, 30.1)	
Type of dwelling			<.0001
Single detached house	67.4 (66.4, 68.4)	30.0 (29.9, 30.1)	
Double, row or terrace, duplex house	10.6 (10.0, 11.3)	29.5 (29.2, 29.8)	
Low-rise or high rise apartment	14.2 (13.6, 14.9)	27.9 (27.7, 28.1)	
Mobile home or other	1.6 (1.4, 1.8)	28.6 (28.2, 29.1)	
Household size			<.0001
Alone	17.5 (16.9, 18.2)	27.8 (27.7, 28.0)	
2 people	45.3 (44.2, 46.4)	29.6 (29.5, 29.7)	
3 or more	31.1 (29.9, 32.3)	30.5 (30.4, 30.7)	
Marital status			<.0001
Married or common-law	69.8 (68.9, 70.7)	30.1 (30.0, 30.2)	
Widowed, separated, or divorced	18.3 (17.7, 19.0)	27.7 (27.5, 27.8)	
Single, or never married	5.8 (5.3, 6.2)	28.8 (28.5, 29.2)	
Cultural/Racial Background			0.0251
Not White	11.4 (10.6, 12.2)	29.3 (29.0, 29.5)	
White	83.0 (82.1, 83.9)	29.6 (29.5, 29.7)	
Immigrant			0.0042
Yes	22.8 (21.9, 23.8)	29.3 (29.1, 29.5)	
No	71.6 (70.6, 72.6)	29.7 (29.6, 29.8)	
County of Birth			<.0001
Canada	70.9 (69.9, 72.0)	29.7 (29.6, 29.8)	
Other North America	1.1 (0.9, 1.3)	29.8 (29.2, 30.4)	
Others	22.3 (21.4, 23.3)	29.3 (29.1, 29.5)	

^a^P-values from t-test or ANOVA testing for differences in mean MOVE. All results weighted using the Statistics Canada proportional sampling scheme and applied Balanced Repeated Replication (BRR) with 500 bootstrap weight variables to obtain the correct standard errors for ANOVA

**Table 3:** Distribution of MOVES across healthcare utilization, health behaviours, or health outcomes

Health Characteristics	Weighted Percentage (95% CI)	Mean MOVES (95% CI)	*p*-value for MOVES^a^
Receipt of Home Care			<.0001
Did not receive home care	81.9 (81.2, 82.7)	30.1 (30.0, 30.2)	
Informal home care only	1.8 (1.6, 2.0)	26.9 (26.6, 27.2)	
Formal home care only	7.6 (7.1, 8.0)	25.8 (25.2, 26.4)	
Both formal and informal home care	2.7 (2.4, 2.9)	23.5 (23.1, 24.0)	
Self-perceived health			<.0001
Excellent	19.4 (18.4, 20.4)	31.2 (31.0, 31.4)	
Very good	32.0 (30.9, 33.0)	30.6 (30.4, 30.7)	
Good	28.3 (27.4, 29.3)	29.1 (29.0, 29.3)	
Fair	10.8 (10.2, 11.4)	26.7 (26.5, 27.0)	
Poor	3.5 (3.1, 3.8)	24.0 (23.5, 24.4)	
Satisfaction with Life Scale			<.0001
Extremely dissatisfied	1.2 (1.0, 1.4)	25.6 (24.8, 26.4)	
Dissatisfied	3.1 (2.7, 3.5)	26.8 (26.2, 27.4)	
Slightly dissatisfied	6.2 (5.6, 6.7)	27.4 (27.0, 27.8)	
Neutral	1.7 (1.4, 1.9)	27.6 (26.9, 28.3)	
Slightly satisfied	13.2 (12.5, 14.0)	28.6 (28.4, 28.8)	
Satisfied	47.3 (46.2, 48.5)	29.9 (29.8, 30.0)	
Extremely satisfied	23.6 (22.7, 24.6)	30.9 (30.7, 31.0)	
Reported having a chronic condition			<.0001
Has at least one chronic condition	73.1 (72.0, 74.2)	29.2 (29.1, 29.3)	
Has no chronic conditions	21.0 (19.9, 22.0)	30.9 (30.8, 31.1)	
Smoking Status			<.0001
Smoker	17.4 (16.5, 18.3)	29.2 (29.0, 29.4)	
Former smoker	45.8 (44.7, 46.9)	29.7 (29.6, 29.9)	
Never smoked	30.7 (29.7, 31.8)	29.5 (29.4, 29.7)	
Drinking Status			<.0001
Regular drinker	58.0 (56.9, 59.1)	30.3 (30.2, 30.4)	
Occasional drinker	16.3 (15.5, 17.2)	29.2 (28.9, 29.4)	
Did not drink in the last 12 months	19.6 (18.8, 20.4)	27.7 (27.5, 27.9)	
Reported having arthritis			<.0001
Yes	26.0 (25.1, 26.9)	28.1 (27.9, 28.2)	
No	67.9 (66.9, 68.9)	30.2 (30.1, 30.3)	
Self-reported BMI			<.0001
Underweight (<18.50)	1.4 (1.2, 1.5)	27.1 (26.4, 27.7)	
Normal weight (18.50–24.99)	37.4 (36.3, 38.5)	29.6 (29.5, 29.8)	
Overweight (25.00–29.99)	36.2 (35.1, 37.3)	29.8 (29.7, 30.0)	
Obese-class I, class 2, class 3 (≥30.00)	21.2 (20.3, 22.2)	29.4 (29.2, 29.6)	
Number of falls (only 65+)			<.0001
No	73.7 (72.7, 74.7)	27.8 (27.7, 27.9)	
One fall	11.5 (10.7, 12.2)	26.4 (26.0, 26.7)	
Two or more falls	6.3 (5.8, 6.8)	24.2 (23.7, 24.7)	
Depressive Symptoms			<.0001
Did not feel depressed or did not lose interest in things	88.9 (88.2, 89.5)	29.7 (29.6, 29.8)	
Felt depressed or lost interest in things	7.7 (7.1, 8.3)	28.1 (27.8, 28.5)	

^a^P-values from t-test or ANOVA testing for differences in mean MOVE. All results weighted using the Statistics Canada proportional sampling scheme and applied Balanced Repeated Replication (BRR) with 500 bootstrap weight variables to obtain the correct standard errors for ANOVA

#### MOVES descriptive statistics

**Fig. 2:**
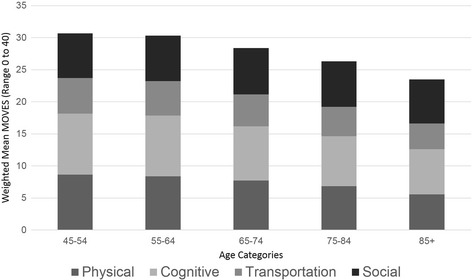
Differences in MOVES Domains by Age

Within the 28,555 adults with complete data to create MOVES, the 10th percentile of MOVES was 24.2 (95% confidence interval (CI) 24.0, 24.4) and the 90th percentile was 34.7 (CI 34.6, 34.9), with a mean of 29.6 (CI 29.5, 29.7). Scores were generally high within each MOVES domain, although differences existed in each domain by age (Figure 2). Out of 10, Canadians scored a mean physical mobility of 8.1 (95% CL 8.1, 8.1), mean cognitive mobility of 9.0 (95% CL 9.0, 9.1), and mean social mobility of 7.1 (95% CL 7.0, 7.1). Over 90% used between one and three transportation modes, giving a mean transportation mobility score of 5.2 (95% CL 5.2, 5.3).

**Fig. 3:**
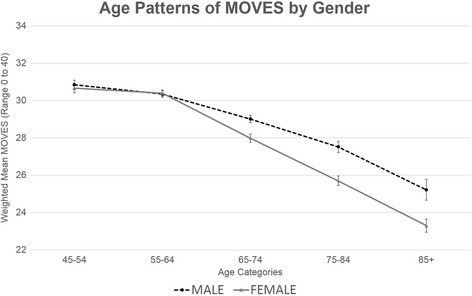
Trend in MOVES with Age, by Gender

MOVES was higher for those who were younger, male, white, better educated, employed, higher income, married, home owners, born in Canada, and living in larger urban areas (Table 2). Higher MOVES was also associated with healthier behaviors and better health outcomes (Table 3). Those with excellent self-perceived health had an average MOVES of 31.2 (CI 31.0, 31.4), compared with those with poor self-perceived health, who had an average MOVES of 24.0 (CI 23.5, 24.4). Lower values for MOVES by age were statistically significantly different for males and females (*p* < 0.001) with females having a steeper decline in mobility across age groups (Figure 3).

MOVES varied across the provinces (*p* < 0.0001) and declines across age groups also varied by province/region (*p* = 0.065, Additional file 1: Fig. S1). Similarly, gender differences in mobility decline differed by province/region (*p* = 0.070, Additional file 2: Fig. S2a and Additional file 3: Fig. S2b).

## Discussion

We used data from a large, population-based study to create a comprehensive measure of mobility, MOVES, that encompasses multiple domains of actualized mobility for mid- to late-life adults living in the community. Grounded in evidence and conceptual frameworks, and refined using input from experts and statistical analysis, MOVES captures the complexity inherent in mobility, including physical, cognitive, social, and transportation domains. Across the representative sample of Canadian older adults, MOVES aligns with expected mobility patterns (higher for those who were younger, higher socioeconomic status, and in better health).

The creation of a holistic mobility score bridges gaps between other classification systems, as it better captures where people go, what they do in their daily lives, and their social connections to others. In contrast to typical clinical measures that focus on physical capacity [6], MOVES provides researchers, practitioners, and policy-makers the opportunity to evaluate actualized mobility more broadly. Particularly noteworthy is our inclusion of transport modes. Older adults out-of-home activity levels decrease with driving cessation [3], and cessation of driving was associated with worse health outcomes [36], although directionality of these associations is unclear. Thus, including both automobile use and transportation alternatives was critical to characterizing older adult mobility. Another novel component of MOVES is its ability to capture social engagement and mobility through tangible social support, sense of belonging, and frequency of participation in community events. Links between social support, health, and overall mortality have been well documented [37, 38], giving further credence to the importance of including social connections and community participation in a mobility score.

The sociodemographic and economic patterns we observed in MOVES align with previous literature on older adult activity [39]. As expected, mobility declines with age. MOVES is higher for men, and declines over age were steeper for women than for men. This differential decline is consistent with reports of ADL in older women [40], and may be due to smaller support networks due to employment patterns, or potential differences in driving. However, gender differences could also result from survivor bias as studies of functional decline show men as less likely to survive [40], possibly resulting in a select group of stronger, more mobile males at older ages. Lower MOVES for those with lower income, education, employment, and home ownership, are consistent with evidence on the role of socioeconomic status in functional status [41], chronic disease [42], and mortality [43]. However, there remains controversy about the mechanisms linking socioeconomic status to mobility [44]. Income and wealth may factor into neighborhood choices, providing fewer options for lower socioeconomic adults. Similarly, educational or occupational differences may afford disparate out of home engagement opportunities or access tools to cope with declines in physical functioning.

Interestingly, we observed higher levels of mobility for Canadians living in larger urban areas. This highlights the need for continued research to differentiate between needs of older adults in rural versus urban centres, and the need to address rural seniors’ health needs [45, 46]. Alternatively, larger-scale geographic patterns by region may be more useful as descriptive distributions of mobility for resource allocation and health care service provision (which is under provincial jurisdiction in Canada). Not surprising, we found those with higher MOVES had better health outcomes, including self-perceived health, life satisfaction, and fewer chronic conditions, normal body weight, fewer depressive symptoms, and fewer falls. These descriptive results are consistent with research findings that life space is associated with health and mortality [5, 47]. Our paper investigated whether trends in our new mobility measure, MOVES, tracked with prevailing literature on mobility patterns. More in-depth analyses should explore the associations between sociodemographic and economic factors, MOVES and health outcomes.

We acknowledge that MOVES has a number of limitations. MOVES was available for only those who answered all included items, assumed that those under 65 have no fear of falling, and items were restricted to those previously measured in CCHS-HA. Other practitioners may benefit from adding in questions on size of social network, cognitive ability to read and understand signage, or other measures related to the conceptual frameworks and domains. We also do not know how MOVES would perform for an institutionalized population. Our example using MOVES to examine mobility patterns also has limitations. First, a Canadian sample may not generalize to other populations. Second, our analyses are not analytic and therefore only show descriptive bivariate patterns between MOVES and the sociodemographic and health variables. Further work would be needed, including age- and other adjustments to examine associations causally. Finally, the sample used was a population-based sample of community dwelling middle-aged and older adults. It does not include people living in institutions, who may have lower mobility. We do not know how well the scale could be used to successfully differentiate between individuals or subgroups with very low levels of mobility. However, MOVES has numerous strengths across potential applications, and fills gaps created by limitations of other classification approaches. MOVES holds utility for researchers working in other population-based survey samples; since MOVES relies on common, pre-existing survey items, others with population surveys can derive a score to study holistic mobility. As such, this score is useful for benchmarking and tracking mobility across large geographic scales. Some MOVES items might not be common to other surveys. Future studies might test whether substituting similar items can be made without compromising the performance of MOVES. Similar to the descriptive analyses we provide, MOVES can be used to ascertain differences across gender, socioeconomic status, geographies and other characteristics. MOVES may also be used in natural experiments to examine changes in mobility with policy shifts or infrastructure investments, although we were unable to test how sensitive MOVES is to change using this cross-sectional sample. Similarly, researchers can use MOVES to understand the association between broad mobility and health outcomes, including self-rated health and overall mortality. Alternatively, MOVES could be used by policy makers and practitioners hoping to better understand mobility. MOVES provides insight on how well older adults are able to engage with their communities, and would enhance discussions around planning for driving cessation and maintaining mobility. Ultimately, MOVES represents the quantitative embodiment of evidence and conceptual frameworks of mobility. By assigning numeric values to these concepts, it further enhances discourse between various stakeholders around supports for older adult mobility and opens new avenues of research.

## Conclusion

Grounded in frameworks and qualitative research that support conceptualizing mobility across physical, cognitive, transport and social domains, this study created a quantitative measurement tool (MOVES) for mobility that encompasses multiple domains. Descriptive data on MOVES in older adults from across Canada followed expected sociodemographic, economic, and health patterns of mobility levels. MOVES appears useful for research, surveillance, evaluation, and interventions around the broad factors influencing mobility in older adults. Future work could use MOVES to examine determinants, consequences and changes in of mobility for older adults across a range of setting and populations.

## Additional files

Additional file 1: Figure S1. Trend in MOVES with age, by Region (PNG 119 kb)

Additional file 2: Figure S2a. Trend in MOVES with age, by Region for Males (PNG 116 kb)

Additional file 3: Figure S2b. Trend in MOVES with age, by Region for Females (PNG 123 kb)

## Abbreviations

ADL: Activities of Daily Living
ANOVA: Analysis of Variance
CCHS-HA: Canadian Community Health Survey – Health Aging
CI: 95% Confidence Interval
CIHR: Canadian Institutes of Health Research
HUI: Health Utilities Index
IADL: Instrumental Activities of Daily Living
ICF: International Classification of Functioning, Disability and Health
MOS: Medical Outcomes Study
MOVES: Mobility Over Varied Environments Scale
NERI: New England Research Institutes
OARS: Older Americans Resources and Services
OMFAQ: OARS Multidimensional Functional Assessment Questionnaire©
PASE: Physical Activity Scale for the Elderly
PCA: Principal Component Analysis
