# Supplementary figures and images for: Developing a comprehensive measure of mobility: mobility over varied environments scale (MOVES)
Source: BMC Public Health. 2017 May 25;17:513. doi: 10.1186/s12889-017-4450-1 (PMC5445376; doi:10.1186/s12889-017-4450-1)

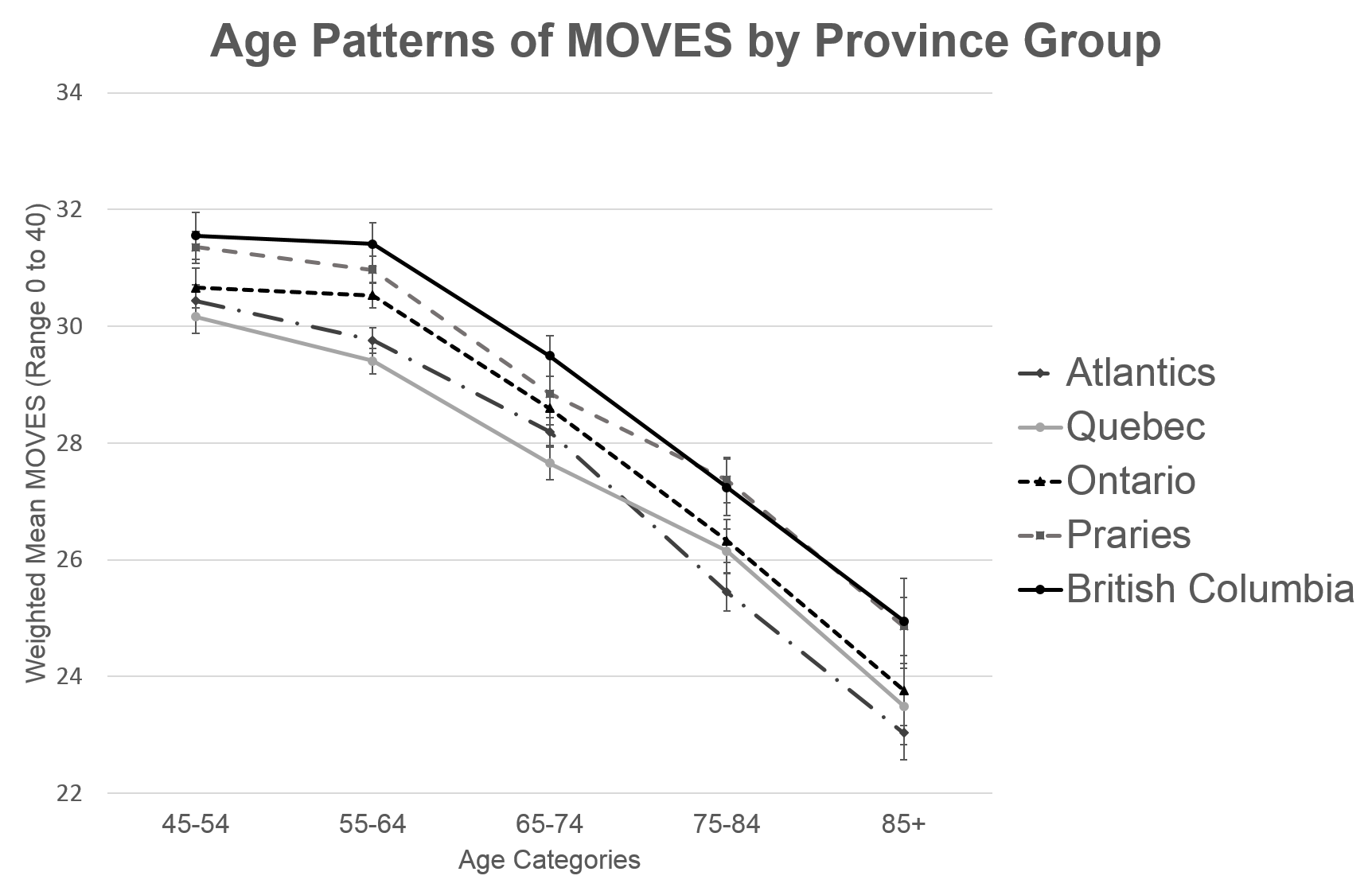

Supplement: Supplementary file 1 — Trend in MOVES with age, by Region (PNG 119 kb) [file 12889_2017_4450_MOESM1_ESM.png]

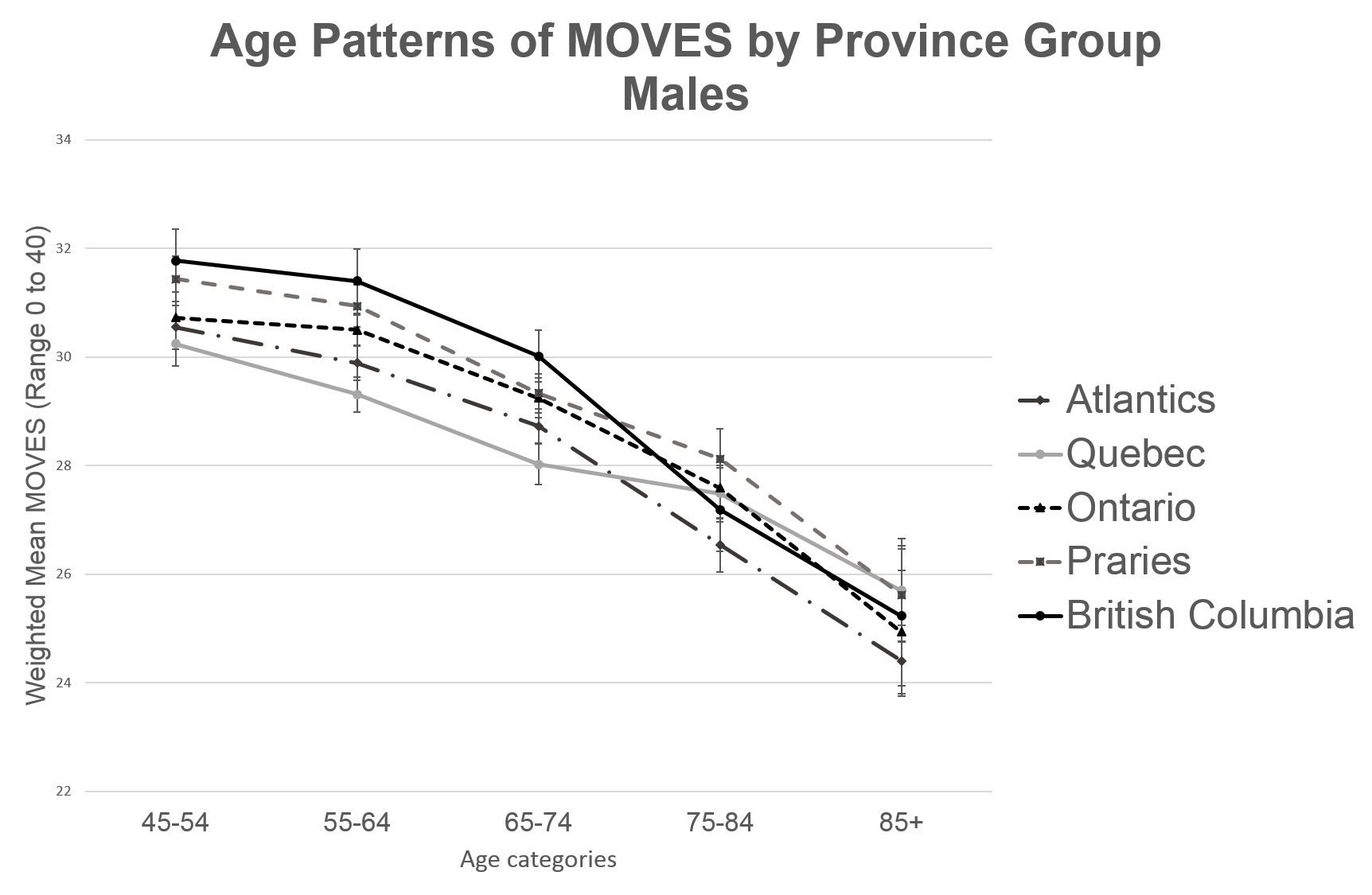

Supplement: Supplementary file 2 — Trend in MOVES with age, by Region for Males (PNG 116 kb) [file 12889_2017_4450_MOESM2_ESM.png]

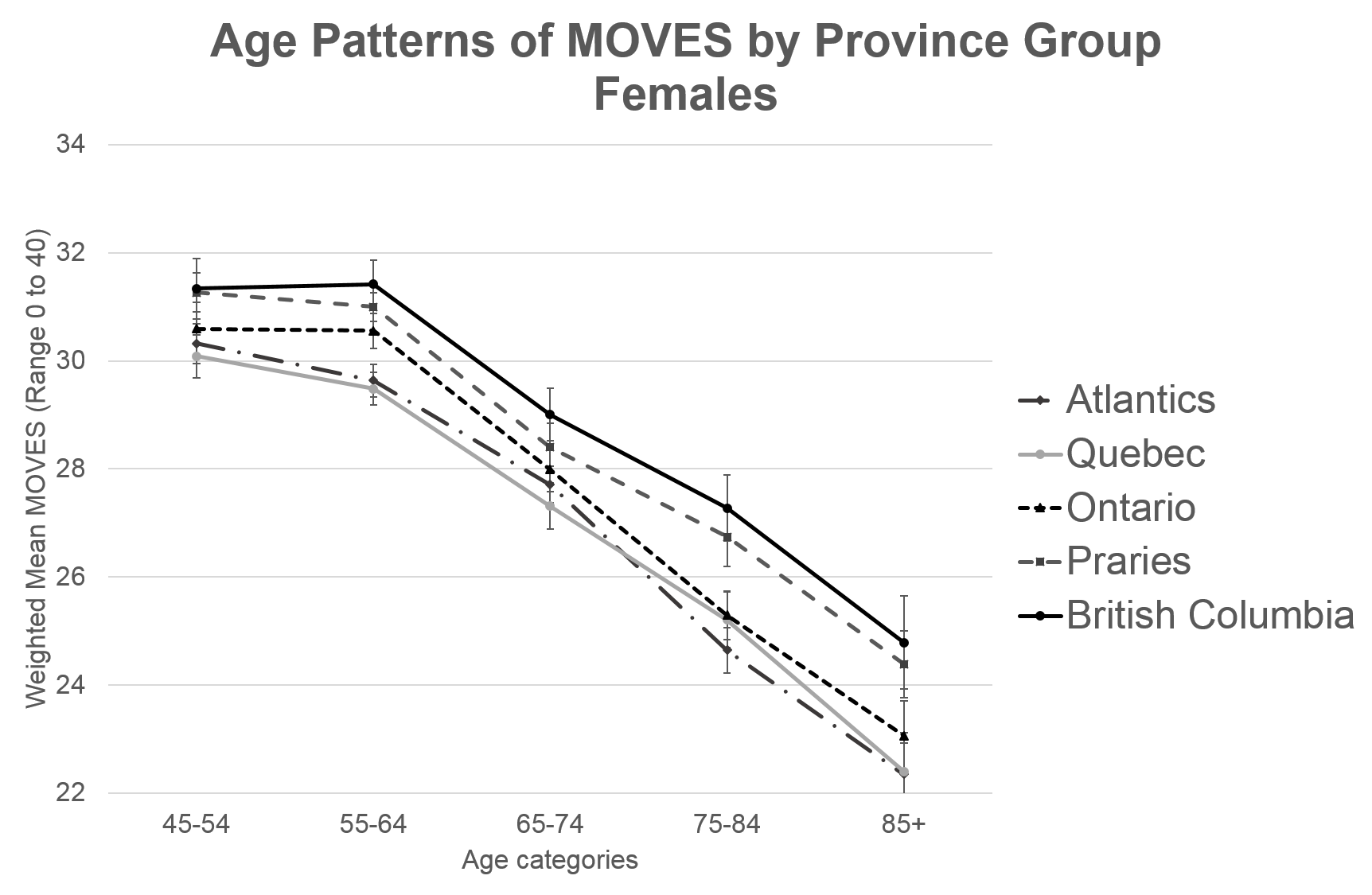

Supplement: Supplementary file 3 — Trend in MOVES with age, by Region for Females (PNG 123 kb) [file 12889_2017_4450_MOESM3_ESM.png]
